# Emergence of Novel Macrolide-Resistant *Mycoplasma pneumoniae* Sequence Type 3 Subclades in Children after COVID-19 Pandemic, Taiwan

**DOI:** 10.3201/eid3211.260439

**Published:** 2026-11

**Authors:** Yu-Chia Hsieh, Chen-Ching Lin, Jia-Wen Wu, Yi-Hsuan Liu, Yi-Jiun Pan, Tzu-Ya Lo, Shian-Sen Shie, Huey-Ling You, Chong-Wei Huang, Yi-Chuan Huang, Tzu-Ching Huang, Yu-Xin She, Yin-Cheng Chen, Shiao-Wen Li

**Affiliations:** Chang Gung Children’s Hospital, Chang Gung Memorial Hospital, Chang Gung University, College of Medicine, Taoyuan, Taiwan (Y.-C. Hsieh, J.-W. Wu, Y.-H. Liu, T.-Y. Lo); School of Medicine, National Yang Ming Chiao Tung University, Taipei, Taiwan (C.-C. Lin, Y.-C. Chen); School of Medicine, College of Medicine, People’s Republic of China Medical University, Taichung, Taiwan (Y.-J. Pan); Chang Gung Memorial Hospital, Taoyuan (S.-S. Shie); Kaohsiung Chang Gung Memorial Hospital, Kaohsiung, Taiwan (H.-L. You); Keelung Chang Gung Memorial Hospital, Keelung, Taiwan (C.-W. Huang, Y.-C. Huang); School of Medicine, College of Medicine, National Yang Ming Chiao Tung University, Taipei (T.-C. Huang); College of Science Department of Life Sciences, National University of Kaohsiung, Kaohsiung (Y.-X. She, S.-W. Li)

**Keywords:** bacteria, antimicrobial resistance, respiratory infections, *Mycoplasma pneumoniae*, macrolide resistance, intensive care unit, central nervous system, whole-genome sequencing, COVID-19, Taiwan

## Abstract

Post–COVID-19 pandemic *Mycoplasma pneumoniae* resurgences have occurred globally and have been particularly severe in Taiwan. We conducted a multicenter study to assess *M. pneumoniae* trends in children. Compared with the 2019 peak, 2024 data revealed a doubling of incidence (546/100,000 population), a 2.2-fold increase in intensive care unit admissions (10.9/100,000 population), and a 13.7-fold surge in central nervous system complications (6.1/100,000 population). In a 2023–2024 prospective cohort (n = 226), intensive care unit admission risk factors were neutrophils >7,000/μL (adjusted odds ratio [aOR] 6.2 [95% CI 1.3–29.9]), platelets >400 × 10^3^/μL (aOR 12.5 [95% CI 2.7–57.9]), pleural effusion (aOR 9.7 [95% CI 1.6–58.1]), and central nervous system involvement (aOR 27.9 [95% CI 4.1–188.3]). Phylogenetic analysis of 698 genomes demonstrated that 4 emerging subclades replaced pre–COVID-19 lineages by mid-2024; 1 subclade was associated with severe outcomes, highlighting evolving resistant, neurotropic lineages and calling for enhanced surveillance.

After the height of the COVID-19 pandemic, *Mycoplasma pneumoniae* respiratory infections did not reemerge as quickly as influenza or respiratory syncytial virus did, likely because of the pathogen’s long incubation period, long periodicity, and slow growth rate (*1*). *M. pneumoniae* activity increased markedly in mid-2023, leading to unprecedented epidemics and varying disease severity across multiple countries worldwide. In parts of Europe and the United States, where macrolide resistance remains <10%, an increase in severe pediatric cases has been reported in Spain, England, and Texas (USA), but not in the Netherlands, Norway, or Denmark (*2*–*5*). France and Switzerland documented high rates of intensive care unit (ICU) admissions among adults (*6*,*7*). In contrast, a highly publicized outbreak in China was notable for an exceptionally high macrolide resistance rate (90%–100%) and a substantial burden of severe disease, primarily affecting children (*8*). Although prolonged fever is frequently reported in macrolide-resistant *M. pneumoniae* infections, an increased risk for complications or ICU admission compared with macrolide-sensitive strains has not been consistently demonstrated (*9*,*10*). Nevertheless, *M. pneumoniae* is increasingly associated with severe clinical manifestations, irrespective of macrolide resistance, although the role of genetic evolution in that trend remains unclear. Timely diagnosis and initiation of appropriate therapy are crucial to reducing disease duration and preventing progression to severe illness requiring intensive care (*7*).

Taiwan has a high prevalence of macrolide-resistant *M. pneumoniae*; resistance rates reached 70% before the COVID-19 pandemic (*10*). In this multisite study, we assessed the effects of the 2023–2024 resurgence among children by comparing the incidence of hospitalization, ICU admission, and neurologic complications across 8 seasons from 2017 through 2024. We also analyzed molecular genetic changes in *M. pneumoniae* and risk factors associated with severe disease in the post–COVID-19 period to better understand pathogen dynamics and inform strategies to combat the disease.

## Materials and Methods

### Incidence Estimation

This study focused on 2 primary objectives. First, we used the Chang Gung Memorial Hospital (CGMH)–based laboratory surveillance system, which spans 4 branches (Linkou, Taipei, Tucheng, and Kaohsiung), representing one of the largest healthcare networks in Taiwan. The study was approved by the Research Ethics Committee of CGMH (approval no. 202102039A3). We identified positive *M. pneumoniae* cases in children (persons <18 years of age) evaluated across outpatient clinics, emergency departments, general wards, and ICUs through respiratory specimens or cerebrospinal fluid (CSF) samples submitted for clinical care. Diagnostic testing for *M. pneumoniae* across the 4 CGMH branches was clinician-driven on the basis of standardized clinical indications. Testing was routinely ordered by attending physicians for pediatric patients with compatible clinical signs and symptoms, such as acute lower respiratory tract infection, persistent fever, or suspected central nervous system (CNS) manifestations. Respiratory specimens collected during January 2017–December 2024 were tested using real-time PCR (*10*) or, starting in 2019, the BioFire Film Array Respiratory Panel (bioMérieux, https://www.biomerieux.com). For patients with suspected CNS involvement, CSF samples were tested using real-time PCR. 

We used data from the National Health Insurance system to estimate the population sizes served by the 4 CGMH branches and then calculated the annual and monthly incidence of *M. pneumoniae* infection. We conducted medical chart reviews to record the diagnosis, hospitalization, ICU admission, and *M. pneumoniae*–related CNS complications, defined as neurologic syndromes occurring in association with *M. pneumoniae* infection, confirmed by PCR detection of *M. pneumoniae* in respiratory or CSF samples, and in the absence of an alternative etiology (*11*). Included manifestations were isolated encephalopathy, encephalitis, brainstem or basal ganglia encephalitis, acute disseminated encephalomyelitis, cerebellitis, Guillain–Barré syndrome, and transverse myelitis. In children demonstrating CNS complications, paired CSF and serum samples were collected on the same day. Paired CSF and serum IgG and albumin concentrations were measured using commercial ELISA kits according to the manufacturer’s instructions. The CSF IgG index was calculated using the followings formula (Figure 5):

**Figure 5:**
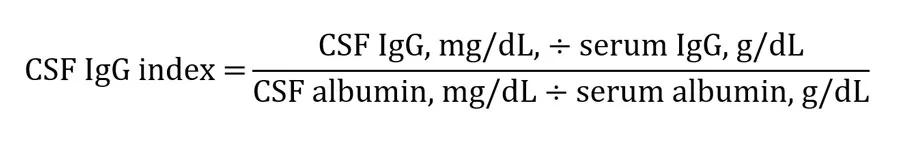
The CSF IgG index was calculated using the followings formula.

### Patients with Risk Factors for ICU Admission

For the second primary objective, we extended a previously initiated prospective study that enrolled children <18 years of age with laboratory-confirmed *M. pneumoniae* infection at CGMH during 2023–2024 (*10*). We excluded children with chronic lung disease, genetic or metabolic disorders, neurologic disease, immunodeficiency, or cancer. Diagnosis required compatible clinical features and confirmation by PCR or culture (*10*). For clinically suspected cases, attending physicians obtained throat swab specimens and forwarded them to the CGMH–Linkou central laboratory, after written informed consent had been secured from patients or their legal guardians. We performed DNA extraction and PCR targeting the 23S rRNA gene as described previously (*10*). We performed multilocus sequence typing (MLST) using the *M. pneumoniae* PubMLST scheme and assigned sequence type (ST) according to the allelic profiles of 8 loci (Appendix 1). We recorded demographic, clinical, and laboratory variables prospectively in a standardized case-report form. We defined severe *M. pneumoniae* infection as admission to an ICU.

### Isolation of *M. pneumoniae* and Determination of MICs

Oropharyngeal swab, pleural fluid, and CSF samples were collected from pediatric patients and cultured as previously described (*10*). For patients with respiratory infection, culture was performed when respiratory specimens were PCR-positive for *M. pneumoniae*. When CNS involvement occurred, CSF culture was performed only when respiratory or CSF specimens were PCR-positive for *M. pneumoniae*. During the 2023–2024 epidemic in Taiwan, a total of 113 *M. pneumoniae* isolates were successfully obtained from 234 specimens (222 respiratory specimens, 8 CSF samples, and 4 pleural fluid samples), consisting of 103 isolates from oropharyngeal swab specimens, 7 isolates from cerebrospinal fluid, and 3 isolates from pleural fluid. MIC testing was performed as previously described (*10*). 

### Whole-Genome Sequencing and Bioinformatic Analysis

We performed whole-genome sequencing on the 113 clinical isolates described previously. We integrated the resulting data with 100 *M. pneumoniae* genomes sequenced in Taiwan during 2018–2020 and published in our previous study (*12*), as well as 485 publicly available global genomes spanning 1960–2024. We quality filtered and assembled raw sequencing reads, then performed separate genome alignments for P1 subtype 1 and P1 subtype 2 against M129 (GenBank accession no. NC_000912.1) and FH (RefSeq assembly accession no. GCF_001272835.1). We generated core-genome single-nucleotide polymorphism alignments using Snippy (https://github.com/tseemann/snippy), then performed phylogenetic reconstruction using Gubbins version 2.4.1 (https://nickjcroucher.github.io/gubbins) (Appendix 1). We identified clade-specific single-nucleotide polymorphisms using a 2-sided 2-proportion test implemented with the prop.test function in R version 4.2.0 (The R Project for Statistical Computing, https://www.r-project.org), applying multiple-testing correction (Appendix 1; Appendix 2).

### Statistical Analysis

We defined incidence rate per 100,000 population as the number of cases divided by estimated population for the year in Taiwan. We defined incidence rate ratio (IRR) as the ratio of incidence rate in year A to incidence rate in year B. We calculated median values and interquartile ranges (IQRs) for continuous variables and used percentages for categorical variables. We compared continuous variables using Mann-Whitney U test; we compared categorical variables using χ^2^ test or Fisher exact test. We used logistic regression analysis to identify factors associated with ICU admission. We included variables with p<0.1 in the univariate analysis in the multivariate analysis. We considered a p<0.05 using a 2-tailed test statistically significant. We performed all statistical analyses using SPSS Statistics version 22.0 (IBM, https://www.ibm.com).

## Results

### Epidemiologic Trends and Clinical Burden of Pediatric *M. pneumoniae* Infection

*M. pneumoniae* cases occurred year-round; monthly positivity rates ranged from 10% to 20% on the basis of 100–200 tests. Large-scale outbreaks were observed in both 2019 and 2024; positivity rates peaked at 40%–50% during June–July. *M. pneumoniae* sometimes showed secondary peaks during January–March (Figure 1, panel A). We analyzed monthly and annual incidences of *M. pneumoniae* infection per 100,000 population by age group: 0–4 years, 5–9 years, 10–17 years, and the combined <18 years cohort. Trends in monthly incidence closely paralleled *M. pneumoniae* detection rates; the highest incidence was consistently seen in children 5–9 years of age, followed by those 0–4 years of age and 10–17 years of age, particularly during late spring and summer (Figure 1, panel B). In 2019, incidence increased markedly across all age groups; a roughly 3-fold increase was observed over 2017 and 2018 (Table 1). Although incidence declined during the COVID-19 pandemic, a substantial resurgence was noted after the pandemic. In 2024, incidence rose ≈1.5- to 2-fold in the 0–4 year (470/100,000 populations) and 5–9 year (986/100,000 populations) age groups and tripled in the 10–17 year age group (313/100,000 populations) compared with 2019 levels (Table 1).

**Figure 1:**
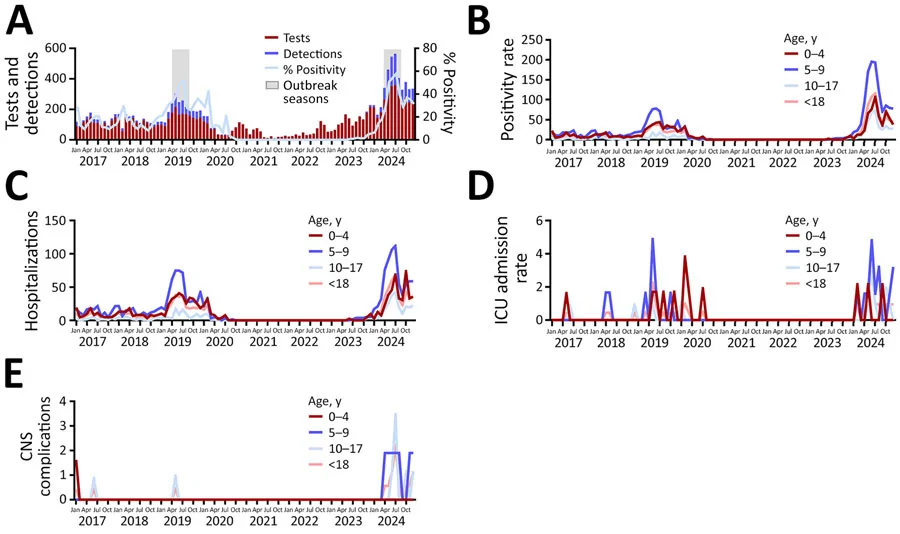
*Mycoplasma pneumoniae*–related incidence among persons <18 years of age in study of emergence of novel macrolide-resistant *M. pneumoniae* sequence type 3 subclades in children after COVID-19 pandemic, Taiwan, 2017–2024 (n = 2,340). A) Monthly numbers of *M. pneumoniae* tests and laboratory-confirmed cases at Chang Gung Memorial Hospital, Taoyuan, Taiwan. B–E) Monthly rates per 100,000 population by age group: B) *M. pneumoniae* positivity; C) *M. pneumoniae*–related hospitalizations; D) *M. pneumoniae*–related ICU admissions; E) *M. pneumoniae*–related CNS complications. CNS, central nervous system; ICU, intensive care unit.

**Table 1:** *Mycoplasma pneumoniae*–related hospitalizations, ICU admissions, and CNS complications among children <18 years of age in study of emergence of novel macrolide-resistant *M. pneumoniae* sequence type 3 subclades in children after COVID-19 pandemic, Taiwan, 2017–2024 (n = 2,340)*

Age group, y	Incidence per 100,000 (no. cases)									2017 vs. 2019			2019 vs. 2024	
	2017	2018	2019	2020	2021	2022	2023	2024		IRR (95% CI)	p value		IRR (95% CI)	p value
*M. pneumoniae* incidence														
<18	87 (206)	90 (204)	284 (635)	55 (112)	0	0	12.8 (28)	546 (1,155)		3.25 (2.78–3.81)	<0.0001		1.92 (1.74–2.11)	<0.0001
0–4	117 (72)	117 (71)	348 (205)	90 (47)	0	0	14.0 (7)	470 (219)		2.99 (2.29–3.91)	<0.0001		1.4 (1.11–1.63)	0.002
5–9	173 (105)	157 (95)	535 (329)	73 (44)	0	0	21.3 (14)	986 (616)		3.09 (2.48–3.85)	<0.0001		1.84 (1.61–2.11)	<0.0001
10–17	26 (29)	36 (38)	98 (101)	23 (21)	0	0	6.8 (7)	313 (320)		3.83 (2.54–5.80)	<0.0001		3.2 (2.55–3.98)	<0.0001
*M. pneumoniae*–related hospitalization														
<18	82 (194)	86 (197)	268 (599)	53 (108)	0	0	12.3 (27)	348 (735)		3.26 (2.77–3.83)	<0.0001		1.30 (1.16–1.44)	<0.0001
0–4	107 (66)	115 (70)	323 (190)	86 (45)	0	0	14.0 (7)	341 (159)		3.02 (2.28–4.00)	<0.0001		1.07 (0.86–1.30)	0.6
5–9	167 (101)	154 (93)	513 (315)	71 (43)	0	0	19.8 (13)	608 (380)		3.07 (2.46–3.85)	<0.0001		1.19 (1.02–1.38)	0.03
10–17	24 (27)	32 (34)	91 (94)	22 (20)	0	0	6.8 (7)	191 (196)		3.83 (2.50–5.88)	<0.0001		2.10 (1.64–2.68)	<0.0001
*M. pneumoniae*–related ICU admission														
<18	0.8 (2)	1.3 (3)	4.9 (11)	2.4 (5)	0	0	0	10.9 (23)		5.8 (1.29–26.2)	0.02		2.2 (1.08–4.53)	0.03
0–4	1.6 (1)	0	8.5 (5)	7.6 (4)	0	0	0	6.4 (3)		5.25 (0.61–44.93)	0.13		0.76 (0.18–3.17)	0.7
5–9	0	3.3 (2)	8.1 (5)	0	0	0	0	19.2 (12)		ND	ND		2.36 (0.83–6.7)	0.11
10–17	0.9 (1)	0.9 (1)	1.0 (1)	1.1 (1)	0	0	0	7.8 (8)		1.1 (0.07–17.6)	0.95		8.04 (1.01–64.3)	0.05
*M. pneumoniae*–related CNS complications														
<18	0.8 (2)	0	0.4 (1)	0	0	0	0	6.1 (13)		0.53 (0.05–5.82)	0.6		13.7 (1.80–104.90)	0.01
0–4	1.6 (1)	0	0	0	0	0	0	0		ND	ND		ND	ND
5–9	0	0	0	0	0	0	0	11.2 (7)		ND	ND		ND	ND
10–17	0.9 (1)	0	0	0	0	0	0	5.9 (6)		1.1 (0.07–17.6)	0.95		6.03 (0.73–50.10)	0.1

*ICU, intensive care unit; IRR, incidence rate ratio; CNS, central nervous system; ND, not done.

*M. pneumoniae*–associated hospitalization incidence and seasonality closely mirrored overall *M. pneumoniae* incidence across all age groups (Table 1; Figure 1, panel C). We further assessed the effects of post–COVID-19 *M. pneumoniae* outbreaks by analyzing ICU admissions (Table 1; Figure 1, panel D) and neurologic complications; an increase was observed specifically in cases of CNS involvement (Table 1; Figure 1, panel E). The incidence of ICU admissions among children <18 years of age increased from 0.8/100,000 population in 2017 to 4.9/100,000 population in 2019 (incidence rate ratio [IRR] 5.8 [95% CI 1.3–26.2]; p = 0.02). Compared with 2019, ICU admission incidence further rose to 10.9/100,000 population in 2024 (IRR 2.2 [95% CI 1.1–4.5]; p = 0.03). Meanwhile, CNS complications showed a striking rise, from 0.4/100,000 population in 2019 to 6.1/100,000 population in 2024 (IRR 13.7 [95% CI 1.8–104.9]; p = 0.01). The incidence of ICU admissions and CNS complications peaked during June–July, consistent with the overall trend of *M. pneumoniae* cases. Of note, the increase in CNS complications was not observed during the 2019 outbreak.

The median age for patients with CNS involvement during the post–COVID-19 period was 8 (IQR 6.8–11.5) years; all patients were >5 years of age (Figure 2; Appendix 1 Table). Absence of respiratory prodrome or respiratory prodrome lasting <7 days was observed in 30.8% (4/13) of patients, whereas 69.2% (9/13) had prodrome lasting >7 days. The most common neurologic syndrome was encephalitis (n = 7), followed by isolated encephalopathy (n = 2), acute disseminated encephalomyelitis (n = 1), transverse myelitis (n = 1), cerebellitis (n = 1), and Guillain-Barré syndrome (n = 1). CSF testing using the BioFire Film Array Meningitis/Encephalitis Panel (bioMérieux) was negative in all 13 patients. CSF culture was performed in 10 patients, yielding *M. pneumoniae* growth in 2 patients with a prodrome of <7 days and in 5 patients with a prodrome of >7 days; all isolates were identified as macrolide-resistant *M. pneumoniae* ST3. CSF *M. pneumoniae* in-house PCR was positive in 1 patient (7.7% [1/13]), in whom 23S rRNA sequencing and MLST identified macrolide-resistant *M. pneumoniae* ST3, although culture was unsuccessful. All 13 patients were treated with doxycycline and corticosteroids; some also received intravenous immunoglobulin, and all had favorable outcomes.

**Figure 2:**
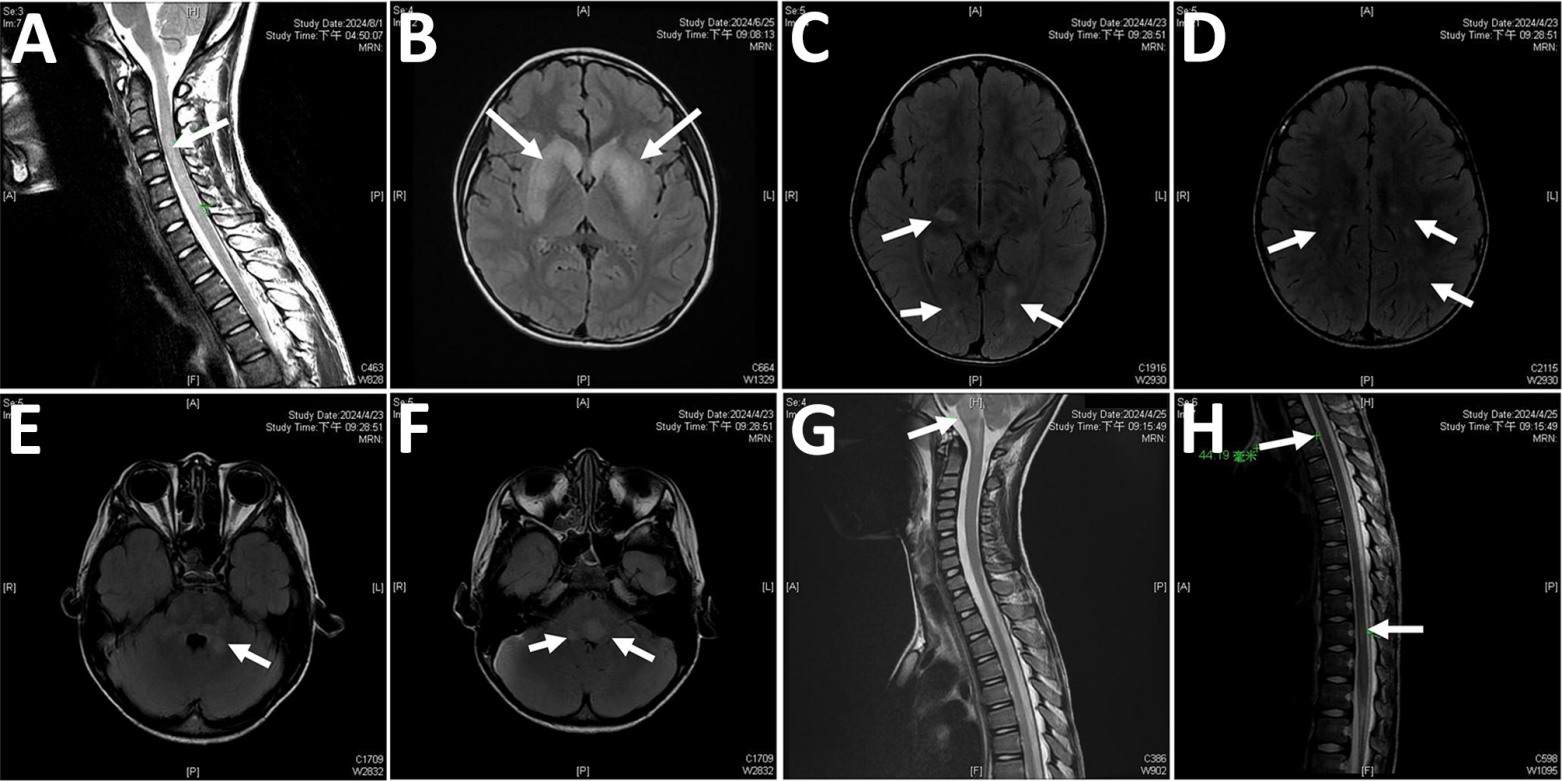
Magnetic resonance imaging (MRI) in selected patients with *M. pneumoniae*–related central nervous system complication in study of emergence of novel macrolide-resistant *M. pneumoniae* sequence type 3 subclades in children after COVID-19 pandemic, Taiwan, 2017–2024. A) Sagittal T2-weighted cervical spine MRI in patient 3, demonstrating a hyperintense lesion extending from C3 to C6 (arrow). B) Axial T2-FLAIR brain MRI of patient 5, showing a focal hyperintense lesion in the bilateral basal ganglia (arrows). C–F) Axial T2-FLAIR brain MRI scans of patient 10, revealing multiple hyperintense lesions involving the subcortical white matter of the bilateral parietal, temporal, and occipital lobes, as well as the midbrain, pons, and middle cerebellar peduncles, findings consistent with acute disseminated encephalomyelitis (arrows). G–H) Sagittal T2-weighted spine MRI of patient 10, indicating hyperintense lesions at the brainstem and at the T3 and T11 spinal levels (arrows).

### Risk Factors for Severe Disease and Alternatives to Macrolide-Resistant *M. pneumoniae* Treatment

A total of 226 patients were prospectively enrolled during the post–COVID-19 period to investigate risk factors associated with ICU admission (Table 2). Of those, 17 patients required ICU care. Compared with non-ICU patients, those admitted to the ICU had higher neutrophil counts and platelet counts and longer hospital stays. CNS complications and pleural effusion were also more frequent among ICU patients. In multivariable analysis, neutrophil count >7,000 cells/μL (odds ratio [OR] 6.2 [95% CI 1.3–29.9]; p = 0.02), platelet count >400 × 10^3^/μL (OR 12.5 [95% CI 2.7–57.9]; p = 0.001), pleural effusion (OR 9.7 [95% CI1.6–58.1]; p = 0.01), and CNS complication (OR 27.9 [95% CI 4.1–188.3]; p = 0.001) remained independently associated with ICU admission (Table 2). We tested upper respiratory tract samples from all patients using the BioFire Respiratory Panel (bioMérieux) to detect respiratory pathogens. Codetection occurred in 62 (27.4%) patients but was not associated with increased complications. MLST and macrolide-resistance mutation analysis revealed that ST3 was the predominant circulating genotype, accounting for 207/226 (91.6%) cases. Macrolide resistance was observed in 219 (96.9%) patients, all of whom carried the A2063G mutation in the 23S rRNA gene. Neither genotype nor resistance profile was associated with clinical outcomes.

**Table 2:** Demographic, clinical, laboratory and radiologic characteristics and risk factors for ICU admission in study of emergence of novel macrolide-resistant *Mycoplasma pneumoniae* sequence type 3 subclades in children after COVID-19 pandemic, Taiwan, 2023–2024 (n = 226)*

Characteristic	ICU admission, n = 17	Non-ICU admission, n = 209	p value	Univariate analysis			Multivariate analysis	
				OR (95% CI)	p value		OR (95% CI)	p value
Median age, y (IQR)	8.5 (7.8–12.0)	8.9 (7.2–10.6)	0.5	1.06 (0.9–1.23)	0.44		–	–
Male sex	9 (52.9)	106 (50.7)	1.0	0.92 (0.34–2.46)	0.86		–	–
Days from symptom onset to hospital admission, median (IQR)	5.0 (1.5–7.0)	6.0 (4.0–7.0)	0.3	0.89 (0.7–0.11)	0.32		–	–
Receiving effective treatment	16 (94.1)	195 (93.3)	1.0	1.15 (0.14–9.3)	0.9		–	–
Days from symptom onset to effective treatment, median (IQR)	7.0 (5.0–7.0)	7.0 (5.0–8.3)	0..3	0.86 (0.7–1.08)	0.2		–	–
Total days with fever, median (IQR)	9 (4–12)	8.0 (6.0–9.0)	0.5	1.06 (0.9–1.3)	0.5		–	–
Median hospital duration, d (IQR)	13.0 (11.5–15.5)	5.0 (1.0–8.0)	<0.001	1.71 (1.4–2.1)	<0.001		–	–
Laboratory characteristic								
Leukocytes, × 10^3^ cells/μL; median (IQR)	11.7 (9.75–13.35)	7.2 (6.0–8.8)	<0.001	1.0 (1.0–1.0)	<0.001		ND	ND
Neutrophil count >7,000 cells/μL	13 (76.5)	31 (14.8)	<0.001	18.7 (5.7–61)	<0.001		6.2 (1.3–29.9)	0.02
Hemoglobin, g/dL; median (IQR)	11.7 (9.7–13.4)	12.3 (11.7–13.1)	0.15	0.77 (0.5–1.06)	0.11		–	–
Platelets, × 10^3^/μL; median (IQR)	451 (265–648)	264 (212–320)	<0.001	1.0 (1.0–1.0)	<0.001		ND	ND
Platelet count >400 × 10^3^/μL	11 (64.7)	19 (9.1)	<0.001	18.3 (6.1–55.1)	<0.001		12.5 (2.68–57.9)	0.001
CRP, mg/L; median (IQR)	45.7 (11–154.4)	26.6 (14.3–62.8)	0.23	1.01 (1.001–1.01)	0.01		1.002 (0.99–1.01)	0.6
AST, U/L; median (IQR)	26.0 (18.5–140.0)	28.0 (23.0–47.3)	0.49	1.001 (1.0–1.003)	0.6			
ALT, U/L; median (IQR)	24.0 (12.0–153.3)	13.0 (9.0–22.0)	0.02	1.001 (1.0–1.003)	0.3			
Codetection	3 (17.6)	59 (28.2)	0.4	0.54 (0.15–1.97)	0.4			
Macrolide resistance	17 (100)	202 (96.7)	1.0	ND	ND			
ST			0.5	ND	ND			
ST3	15 (88.2)	192 (91.9)						
ST14	2 (11.8)	12 (5.7)						
Others	0	5 (2.4)						
Chest radiography findings								
Increased infiltration	8 (47.1)	71 (34)	0.3	1.7 (0.6–4.7)	0.28			
Patchy consolidation	9 (52.9)	138 (66)	0.3	0.58 (0.2–1.57)	0.28			
Pleural effusion	5 (29.4)	14 (6.7)	0.008	5.8 (1.8–18.8)	0.003		9.96 (1.6–58.1)	0.01
CNS complication				21.4 (5.07–89.9)	<0.001		27.9 (4.1–188.3)	0.001

*Values are no. (%) except as indicated. AST, aspartate aminotransferase; ALT, alanine aminotransferase; CNS, central nervous system; CRP, Cc-reactive protein; ICU, intensive care unit; ND, not done; OR, odds ratio; ST, sequence type.

Among 219 macrolide-resistant *M. pneumoniae* strains, ST3 predominated (207 [94.5%]), followed by ST14 (10 [4.6%]), ST11 (1 [0.45%]), and ST44 (1 [0.45%]). Among 7 macrolide-susceptible *M. pneumoniae* strains, we identified ST14 (4 [57.1%]), ST2, ST3, and ST17 (each 1 [14.3%]). We determined MIC ranges, MIC_50_ (MIC at which 50% of antimicrobial is inhibited), and MIC_90_ (MIC at which 90% of antimicrobial is inhibited) values for 10 antimicrobial agents in 109 culturable macrolide-resistant isolates (Table 3). Doxycycline demonstrated the lowest MIC_50_ (<0.03 mg/L) and MIC_90_ (0.03 mg/L), and no isolates showed resistance to levofloxacin or moxifloxacin according to Clinical and Laboratory Standards Institute guidelines (*13*).

**Table 3:** Macrolide-resistant *Mycoplasma pneumoniae* strains and antimicrobial susceptibility to 10 agents in study of emergence of novel macrolide-resistant *M. pneumoniae* sequence type 3 subclades in children after COVID-19 pandemic*

Antimicrobial agent	Macrolide-resistant *M. pneumoniae* with A2063G mutation, n = 109		
	MIC_50_	MIC_90_	MIC range
Macrolide			
Erythromycin	64	128	16–256
Azithromycin	16	32	1–128
Clarithromycin	32	128	2–128
Quinolones			
Levofloxacin	0.25	0.5	0.03–0.5
Moxifloxacin	0.03	0.06	<0.03–0.06
Ciprofloxacin	0.5	1	0.06–1
Tosufloxacin	0.06	0.125	0.03–0.25
Tetracyclines			
Doxycycline	<0.03	0.03	<0.03–0.125
Minocycline	0.03	0.06	<0.03–0.125
Omadacycline	0.06	0.125	0.03–0.125

*MIC_50_, MIC at which 50% of isolates are inhibited; MIC_90_, MIC at which 90% of isolates are inhibited.

### Shifts in *M. pneumoniae* Genomic Structure during Postpandemic Period

To identify the specific genomic variations associated with the postpandemic resurgence of ST3, we analyzed a total of 698 *M. pneumoniae* genomes. That dataset consisted of 100 prepandemic isolates from Taiwan (2018–2020), 113 isolates collected during the 2023–2024 outbreak, and 485 publicly available global genomes spanning 1944–2024 across 4 continents (Figure 3, panel A). Phylogenetic analysis identified 5 major clades; no novel high-level clades emerged during the post–COVID-19 resurgence. However, we observed a striking regional shift in lineage dominance. Across East Asia, ST3 (T1–2) and ST14 (T2–2) were the predominant clades during the postpandemic period. ST3 accounted for 89.4% (101/113) of postpandemic isolates in Taiwan and 81.4% (232/285) in China.

**Figure 3:**
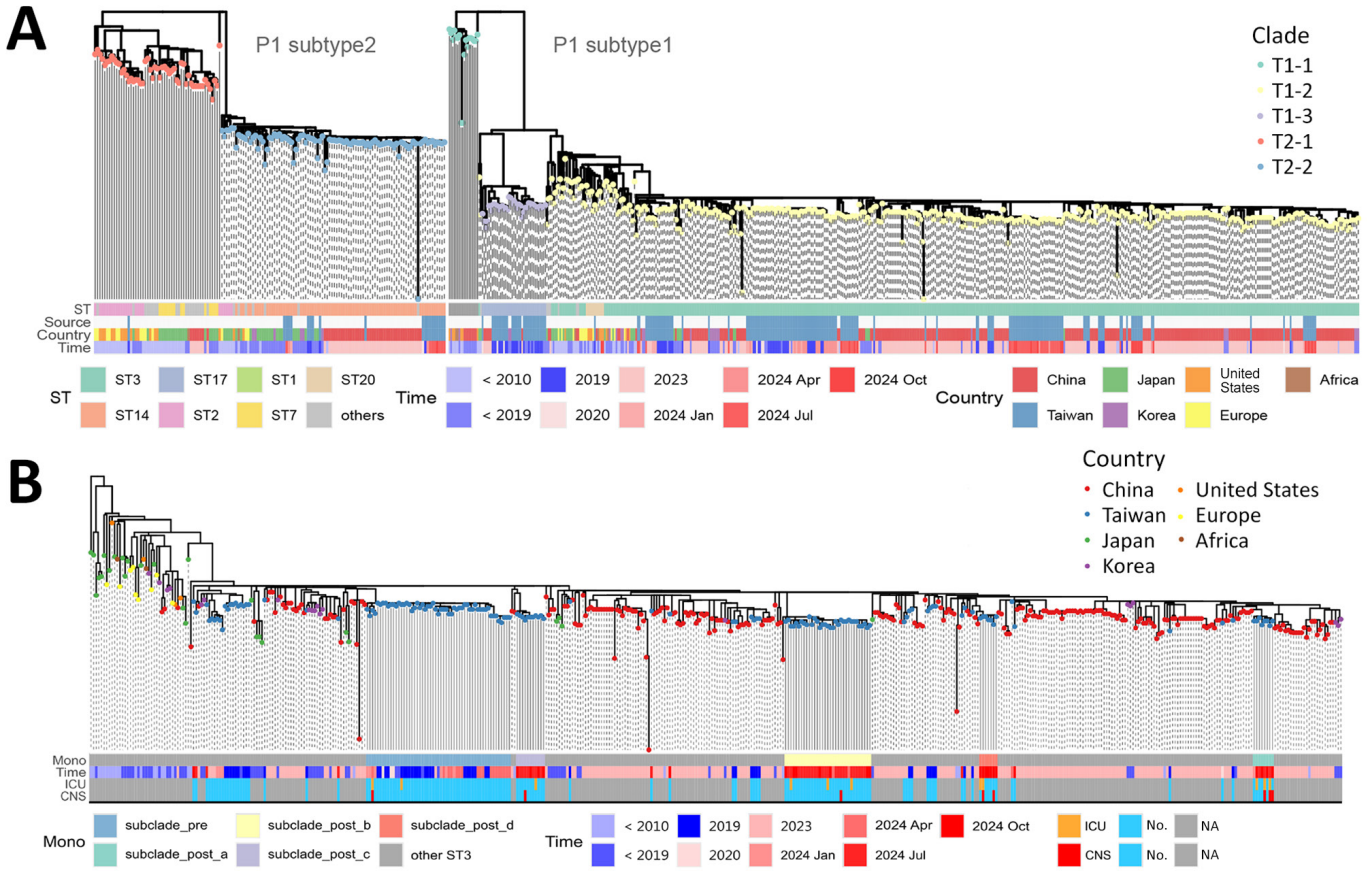
Phylogenomic evolution of *Mycoplasma pneumoniae* in study of emergence of novel macrolide-resistant *M. pneumoniae* ST3 subclades in children after COVID-19 pandemic, Taiwan, 2017–2024. A) Global phylogenetic tree showing evolutionary relationships among all sequenced strains. Nodes are colored by clade according to our previous study (*12*). Annotation bands indicate sequence type, data source, country, and sampling time. For 2024, isolates were grouped into quarterly intervals (January–March, April–June, July–September, and October–December) to provide greater temporal resolution during the outbreak. B) Phylogeny of ST3 strain. Nodes are colored by country, with annotation bands indicating monophyletic subclades (Mono), sampling time, ICU admission, and CNS complications. CNS, central nervous system; ICU, intensive care unit; ST, sequence type.

Given that ST3 represented ≈80% of circulating strains in East Asia, we examined the phylogenetic structure within this lineage (Figure 3, panel B). Root-to-tip regression demonstrated a robust temporal signal (Pearson correlation coefficient = 0.573) (Figure 4, panel A), supporting clock-like accumulation of mutations and reliable reconstruction of evolutionary trajectories. ST3 isolates from Taiwan segregated into 5 distinct monophyletic groups that were phylogenetically separate from dominant ST3 strains from China, indicating independent regional diversification rather than simple importation.

**Figure 4:**
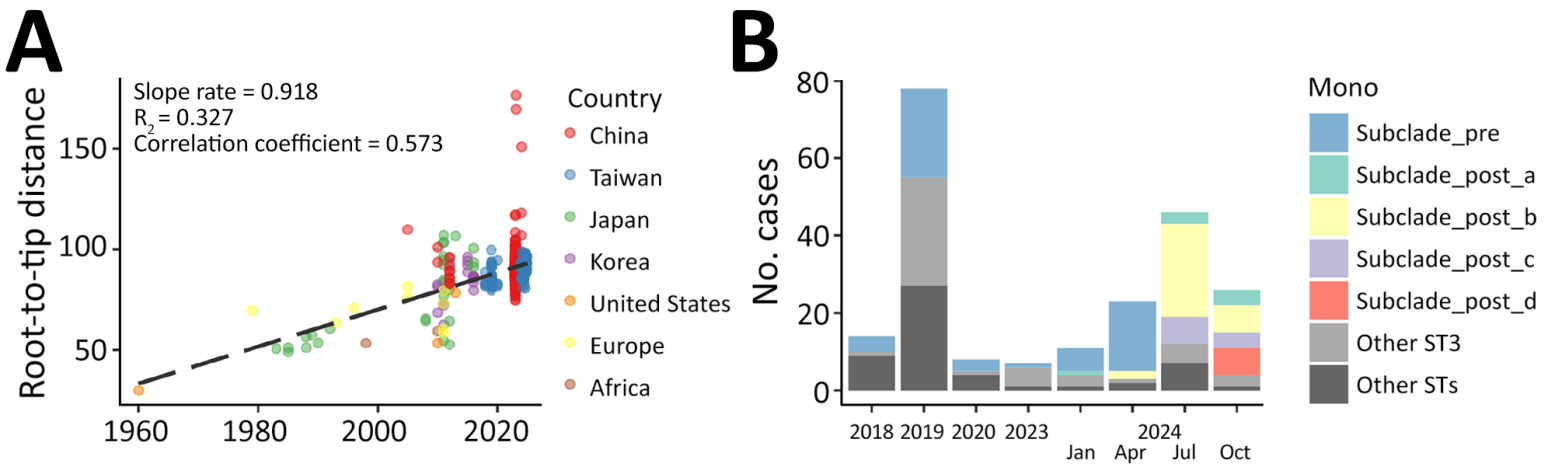
Temporal prevalence of *Mycoplasma pneumoniae* in study of emergence of novel macrolide-resistant *M. pneumoniae* sequence type 3 subclades in children after COVID-19 pandemic, Taiwan, 2017–2024. A) Temporal signal in ST3 genomes evaluated by linear regression of root-to-tip genetic distances against sampling year for global isolates collected during 1960–2024, indicating molecular clock consistency. B) Temporal prevalence of ST3 subclades among persons <18 years of age, Taiwan, 2018–2024. Isolates collected in 2024 are shown at quarterly intervals. ST, sequence type.

A preexisting Taiwan lineage (subclade_pre), which accounted for 50% of prepandemic ST3 isolates (30/60), persisted into 2023 and early 2024 and accounted for 61% of ST3 cases (25/41) in the first half of the year. However, that lineage rapidly declined and was completely replaced in the second half of 2024 (Figure 4, panel B), demonstrating clear within-lineage turnover. In contrast, 4 newly emerged ST3 lineages (subclade_post_a–d) (Figure 3) progressively appeared and had largely replaced subclade_pre by mid-2024. Those postpandemic subclades accumulated 7, 14, 15, and 9 lineage-specific mutations (Appendix 2 Table 1). That clade replacement temporally coincided with the peak in *M. pneumoniae*–related hospitalizations, ICU admissions, and CNS complications, and accounted for most severe cases. Subclade_post_b underwent rapid expansion and became dominant in late 2024 (43.1% of isolates). The lineage was characterized by lineage-specific mutations in MPN_RS01335 and MPN_RS01875 (Appendix 2 Table 1). Of note, subclade_post_a exhibited significantly higher percentages of ICU admissions and CNS complications (both 37.5%) than other clades (p = 0.015 for ICU admissions and p = 0.00061 for CNS complications by the χ^2^ test). That lineage was characterized by lineage-specific mutations in MPN_RS01790, MPN_RS04325, and MPN_RS02200 (Appendix 2 Table 1).

## Discussion

The 2024 resurgence of *M. pneumoniae* in Taiwan was characterized by the predominance of macrolide-resistant ST3 strains, continuous genomic diversification, and a notable increase in ICU admissions and neurologic complications correlated with CNS invasion. In Denmark, where *M. pneumoniae* is a notifiable disease, surveillance data revealed post–COVID-19 incidence rates comparable to those observed in our study; 500–600 PCR-confirmed cases and 200–300 hospitalizations occurred per 100,000 children <18 years of age in 2024 (*14*). A rise in *M. pneumoniae*–associated rash and mucositis was noted, without a corresponding increase in ICU admissions (*14*). In China, the 2023 surge of *M. pneumoniae* infections involved severe disease, including dyspnea requiring bronchoalveolar lavage, necrotizing pneumonia with pulmonary thrombosis, and parapneumonic effusion in 25% of cases (*15*,*16*). Those infections were associated with the continued circulation of macrolide-resistant ST3 and ST14 strains, which clustered with isolates detected during 2019–2022 on phylogenetic analysis. ST3 demonstrated genomic enrichment in virulence, signaling, and metabolism-related genes compared with ST14, potentially explaining its greater clinical effects and predominance (*8*,*17*). Consistent with previous reports from China, Japan, and South Korea (*8*,*18*,*19*), our comparative genomic analysis demonstrated that ST3 (T1–2) remained the predominant lineage during the postpandemic resurgence in Taiwan and China, indicating its continued widespread circulation across East Asia. In adults, the frequent atypical radiological manifestation of alveolar consolidation, which contributed to delays in effective treatment, was associated with a 31.7% ICU admission rate during the 2023–2024 *M. pneumoniae* epidemic in France (*7*). Regional differences in the clinical spectrum and severity of *M. pneumoniae* infection might result from challenges in initiating appropriate treatment, especially in areas with high macrolide resistance, compounded by viral co-infections, and diminished innate immune priming because of prolonged pandemic restrictions (*2*,*8*,*20*). However, the increase in *M. pneumoniae*–related CNS complications, typically occurring in parallel with community outbreaks, has been seldom addressed after the COVID-19 pandemic and was not observed during the 2019 *M. pneumoniae* wave in Taiwan (*10*).

The pathogenic mechanisms of *M. pneumoniae* involve direct damage to the respiratory epithelium through toxic molecules including hydrogen peroxide, hydrogen sulfide, the community-acquired respiratory distress syndrome toxin (*21*), and tissue injury resulting from excessive immune response (*22*). In addition to respiratory tract infections, *M. pneumoniae* can cause extrapulmonary manifestations; encephalitis is one of the most severe (*22*). *M. pneumoniae* infection has been identified in 5%–10% of pediatric encephalitis cases; up to 60% of affected patients develop long-term neurologic sequelae (*22*). *M. pneumoniae*–related encephalitis involves 2 main pathogenic mechanisms: direct CNS invasion and postinfectious immune-mediated injury (*23*). *M. pneumoniae* DNA is typically detectable in 50%–80% of CSF samples within 1 week of respiratory symptoms or in cases without prodrome (*11*,*24*). When prodrome lasts >7 days, *M. pneumoniae* DNA is detected in only 7%–14% of CSF samples, supporting the notion that disease at that stage is predominantly immune-mediated (*11*,*24*). Among 13 patients with CNS complications, respiratory multiplex PCR identified *M. pneumoniae* as the sole pathogen in 11 (84.6%) patients. Although CSF PCR for *M. pneumoniae* was positive only in 1 case, CSF cultures were performed in 10 patients and yielded growth in 7 (70%) patients, irrespective of prodrome duration. The discrepancy between PCR-negative and culture-positive results likely reflects low bacterial load (<400 copies/mL) (*24*). Of note, those findings reduce the likelihood of alternative infectious etiologies and suggest a neuroinvasive potential of macrolide-resistant *M. pneumoniae* ST3 strains; some cases show evidence of postinfectious immune response, as indicated by an elevated CSF IgG index. During the 2019 *M. pneumoniae* epidemic wave in Taiwan, attention focused on the use of doxycycline to manage *M. pneumoniae* infections, given the high prevalence of macrolide resistance (*10*). In the 2023–2024 wave, doxycycline was adopted as the first-line agent for most patients. The favorable outcomes observed in our patients might be attributed to the synergistic effect of doxycycline’s reliable CNS penetration and the early, aggressive use of immunomodulatory therapy. The American Academy of Pediatrics considers doxycycline safe for children <8 years of age when prescribed for <21 days (*25*) and also recognizes the role of fluoroquinolones, which are available in intravenous formulations and achieve favorable CNS penetration, in the management of multidrug-resistant infections (*26*). In our study, macrolide-resistant isolates remained uniformly susceptible to tetracyclines and fluoroquinolones, confirming their value as essential therapeutic options for macrolide-resistant *M. pneumoniae*. In regions characterized by high rates of macrolide resistance, doxycycline, levofloxacin, and moxifloxacin might serve as preferred first-line therapeutic options.

*M. pneumoniae* has a reduced genome of ≈800 kb that has remained relatively stable across time and geography. Sequence variations are predominantly located in the antigenic regions of P1 and P40/P90, which together form the Nap transmembrane complex, responsible for host antibody recognition and binding to sialic acid oligosaccharides of target host cells (*27*). Our previous study demonstrated that ST3 exhibits high homologous recombination capacity and extensive genomic diversity, which might explain its global epidemiologic success despite selective pressure from widespread macrolide use (*12*). In the post–COVID-19 era in Taiwan, that lineage appears to have undergone further sustained expansion through specific adaptive changes. The lack of association between the broad ST3 genotype and clinical outcomes suggested that finer-scale genomic variations within the dominant lineage might be responsible for the observed surge in severe cases. Our genomic analysis identified lineage-specific mutations in several genes that might warrant further investigation. Subclade_post_b, which became predominant in late 2024, was characterized by mutations in MPN_RS01335, encoding thioredoxin-disulfide reductase, and MPN_RS01875, encoding an ABC transporter permease. Of note, subclade_post_a was significantly associated with increased disease severity, accounting for disproportionately higher rates of ICU admissions and CNS complications. That lineage was characterized by mutations in MPN_RS02200, encoding a dihydrolipoamide acetyltransferase family protein; MPN_RS04325, encoding a DUF16 domain-containing protein; and MPN_RS01790, encoding an APC family permease.

The first limitation of this study is that the rise in severe cases could partly reflect the expanded scale of the total surge, and the clinical effects of the identified mutations require further functional validation. The hospital-based cohort might overrepresent severe cases and underestimate community prevalence. PCR cannot fully distinguish active infection from asymptomatic carriage; however, all patients were symptomatic and tested after physician assessment, reducing that concern. Strengths of the study include its multicenter design, comprehensive assessment of incidence and risk factors for severe disease, detailed antimicrobial susceptibility testing, and genome analysis associated with severe and neuroinvasive manifestations. 

In summary, our findings indicate that *M. pneumoniae* clinical effects extend beyond self-limiting pneumonia and highlight the ongoing genomic diversification of ST3 during the postpandemic resurgence. Continued genomic surveillance is essential to monitor emerging variants and guide public health strategies and therapeutic interventions.

Appendix 1 Additional information from study of emergence of novel macrolide-resistant *Mycoplasma pneumoniae* sequence type 3 subclades in children after COVID-19 pandemic.

Appendix 2 Additional data from study of emergence of novel macrolide-resistant *Mycoplasma pneumoniae* sequence type 3 subclades in children after COVID-19 pandemic.
