# Supplementary material for: Emergence of Novel Macrolide-Resistant Mycoplasma pneumoniae Sequence Type 3 Subclades in Children after COVID-19 Pandemic, Taiwan
Source: Emerg Infect Dis. 2026 Nov;32(11):1827–37. doi: 10.3201/eid3211.260439 (PMC13715894; doi:10.3201/eid3211.260439)
Supplement: Supplementary file 1 [file 26-0439-Techapp-s1.pdf]

Article DOI: https://doi.org/10.3201/eid3211.260439

EID cannot ensure accessibility for supplementary materials supplied by authors. Readers who have difficulty accessing supplementary content should contact the authors for assistance.

Emergence of Novel Macrolide-Resistant *Mycoplasma pneumoniae* Sequence Type 3 Subclades in Children after COVID-19 Pandemic, Taiwan

Appendix 1

Supplementary Methods

Determination of Minimal Inhibitory Concentrations (MICs)

MICs were evaluated in triplicate for 10 antimicrobial agents (Sigma-Aldrich, St. Louis, MO, USA) against all viable MP isolates, strictly adhering to Clinical and Laboratory Standards Institute (CLSI) M43-A guidelines. The panel encompassed three macrolides (erythromycin, azithromycin, clarithromycin), four fluoroquinolones (levofloxacin, moxifloxacin, ciprofloxacin, tosufloxacin), and three tetracyclines (doxycycline, minocycline, omadacycline). Serial 2-fold drug dilutions were prepared in 96-well microdilution plates and inoculated with a final bacterial density of 104–105 CFU/mL. Microplates were incubated at 37°C in a 5% CO_2 atmosphere for 5 to 14 days. Endpoints were recorded upon initial color transition (red to yellow) in the growth control wells. MP reference strain M129 (ATCC 29342) served as a quality control for drug susceptibility, while drug-free wells containing medium or solvent alone were maintained as sterility and negative controls.

Multilocus Sequence Typing (MLST)

The *M. pneumoniae* MLST scheme included eight loci: ppa (256 bp), pgm (1,072 bp), gyrB (429 bp), gmk (394 bp), glyA (676 bp), atpA (796 bp), arcC (570 bp), and adk (473 bp).

Sequence types were assigned according to the allelic profiles in the PubMLST database (https://pubmlst.org/mpneumoniae/).

Whole-Genome Sequencing (WGS) and bioinformatics analysis

113 clinical isolates cultured 10^9 cfu amount to approach library preparation for sequencing on the Illumina Nova platform generating paired-end reads of 150 bp. BBDuk v. 38.79, a tool included in the BBMap package (https://sourceforge.net/projects/bbmap/) was used to remove both adapters and low-quality sequences. Followed by de novo assembly was performed using SPAdes v. 3.15.5 (*1*) with default settings. We performed further quality control of assemblies with QUAST v. 5.2.0 (*2*). The draft genomes sequenced in this study have been deposited in GenBank, and their accession numbers are listed in Appendix 2 Table 1. Genotyping was performed using Multilocus sequence typing (MLST) to identify sequence types (STs) to better understand the molecular epidemiology of MP by PubMLST.

SNPs specific to each clade were identified using a two-proportion test. For each genomic position, the most prevalent nucleotide across all isolates was defined as the reference allele. The proportion of isolates carrying the reference allele in the target clade was compared with that in all other isolates using a two-sided two-proportion test implemented with the prop.test function in the R statistical environment (version 4.2.0; R Foundation for Statistical Computing, Vienna, Austria). Sites with a Benjamini–Hochberg-adjusted p value $<1 \times 10^{-5}$ and an alternative allele prevalence >95% within the target clade were considered clade-specific mutations.

References

1. Nurk S, Meleshko D, Korobeynikov A, Pevzner PA. metaSPAdes: a new versatile metagenomic assembler. Genome Res. 2017;27:824–34. PubMed https://doi.org/10.1101/gr.213959.116

2. Mikheenko A, Prjibelski A, Saveliev V, Antipov D, Gurevich A. Versatile genome assembly evaluation with QUAST-LG. Bioinformatics. 2018;34:i142–50. PubMed https://doi.org/10.1093/bioinformatics/bty266

Appendix 1 Table. *Mycoplasma pneumoniae*-associated central nervous system complications, clinical symptoms, laboratory findings, diagnoses and treatments, Taiwan, 2023–2024 (n = 13)*

				Oropharyngeal swab					Blood			CSF MP				Treatment		
Pt. no.	Age/Sex	Onset symptom	Respiratory prodrome	MP	MLST	Genotypic resistance	Codetection	CSF leukocyte/protein	serology IgM/IgG	CSF IgG index	Diagnosis	PCR	Culture	MLST	Genotypic resistance	Doxycycline plus steroid	IVIG	Chest x-ray
1†	12/F	limbs weakness	14 d	+	ST3	R	-	0/45.6	+/+	0.43	Guillain Barre syndrome	-	ND	ND	ND	+	-	infiltration
2	13.8/M	lethargy	no	+	ST3	R	-	305/122.6	ND	1.29	Encephalitis	-	+	ST3	R	+	+	ND
3	12.9/M	unsteady gait	10 d	+	ST3	R	-	95/52.7	+/+	0.69	Transverse myelitis	-	+	ST3	R	+	+	consolidation
4‡	9/F	seizure	21 d	+	ST3	R	-	38/22.4	−/−	0.66	Encephalitis	-	+	ST3	R	+	+	infiltration
5	8.5/M	ataxia, seizure	7 d	+	ST3	R	adenovirus	0/18.2	+/+	0.59	Basal ganglia encephalitis	+	-	ST3	R	+	+	infiltration
6	10.4/M	seizure	2 d	+	ND	ND	-	2/22.2	ND	ND	Isolated encephalopathy	-	ND	ND	ND	+	-	infiltration
7	13/M	strange behavior	4 d	+	ND	ND	-	1/18.7	ND	0.56	Isolated encephalopathy	-	-	ND	ND	+	-	infiltration
8	11.8/M	unsteady gait	10 d	+	ND	ND	-	80/57.8	+/+	0.55	Encephalitis	-	ND	ND	ND	+	+	infiltration
9	7.9/M	seizure	5 d	+	ST3	R	-	36/25	ND	0.61	Encephalitis	-	+	ST3	R	+	+	normal
10†	8.4/M	diplopia, dysphagia	21 d	+	ST3	R	-	115/178.5	+/+	1.65	ADEM	-	-	ND	ND	+	+	infiltration
11§	10/F	unsteady gait	7 d	+	ST3	R	-	41/28.9	+/−	0.63	Cerebellitis	-	+	ST3	R	+	-	consolidation
12§	6/F	lethargy	8 d	+	ST3	R	rhinovirus / enterovirus	85/56.2	+/+	1.21	Encephalitis	-	+	ST3	R	+	-	consolidation
13	7.8/M	lethargy	9 d	+	ST3	R	-	33/40.1	+/+	1.17	Encephalitis	-	+	ST3	R	+	-	infiltration

*ADEM, acute disseminated encephalomyelitis; CSF, cerebrospinal fluid; MLST, multilocus sequence typing; MP, *Mycoplasma pneumoniae;* ND, not done; ST, sequence type
†GM2/GM3 Ab detected in patient 1; MOG Ab detected in patient 10.
‡Patient 4 had a history of epilepsy, while the remaining 12 patients were previously healthy.
§CSF mycoplasma IgM show positive, not performed in other patients.
